# Incidental Memory of Younger and Older Adults for Objects Encountered in a Real World Context

**DOI:** 10.1371/journal.pone.0099051

**Published:** 2014-06-18

**Authors:** Xiaoyan Qin, Tiana M. Bochsler, Alaitz Aizpurua, Allen M. Y. Cheong, Wilma Koutstaal, Gordon E. Legge

**Affiliations:** 1 Department of Psychology, University of Minnesota, Minneapolis, Minnesota, United States of America; 2 Faculty of Psychology, University of the Basque Country, Donostia-San Sebastián, Spain; 3 School of Optometry, The Hong Kong Polytechnic University, Hong Kong; University of Melbourne, Australia

## Abstract

Effects of context on the perception of, and incidental memory for, real-world objects have predominantly been investigated in younger individuals, under conditions involving a single static viewpoint. We examined the effects of prior object context and object familiarity on both older and younger adults’ incidental memory for real objects encountered while they traversed a conference room. Recognition memory for context-typical and context-atypical objects was compared with a third group of unfamiliar objects that were not readily named and that had no strongly associated context. Both older and younger adults demonstrated a typicality effect, showing significantly lower 2-alternative-forced-choice recognition of context-typical than context-atypical objects; for these objects, the recognition of older adults either significantly exceeded, or numerically surpassed, that of younger adults. Testing-awareness elevated recognition but did not interact with age or with object type. Older adults showed significantly higher recognition for context-atypical objects than for unfamiliar objects that had no prior strongly associated context. The observation of a typicality effect in both age groups is consistent with preserved semantic schemata processing in aging. The incidental recognition advantage of older over younger adults for the context-typical and context-atypical objects may reflect aging-related differences in goal-related processing, with older adults under comparatively more novel circumstances being more likely to direct their attention to the external environment, or age-related differences in top-down effortful distraction regulation, with older individuals’ attention more readily captured by salient objects in the environment. Older adults’ reduced recognition of unfamiliar objects compared to context-atypical objects may reflect possible age differences in contextually driven expectancy violations. The latter finding underscores the theoretical and methodological value of including a third type of objects–that are comparatively neutral with respect to their contextual associations–to help differentiate between contextual integration effects (for schema-consistent objects) and expectancy violations (for schema-inconsistent objects).

## Introduction

In the real world, objects are always located within a spatial context, and generally appear with other objects. Previous studies have documented the importance of contextual information to visual processing and perceptual memory, and have shown that the influence of context on recognition may depend on multiple factors related to stimulus features, perceiver characteristics, and processing circumstances [1]–[3]. However, with comparatively few exceptions [4]–[6] investigations have most often involved the viewing of 2-D or computerized images of objects or scenes–situations that do not fully reflect the challenges of vision in the real world. Examining performance under natural viewing conditions, including during realistic movements through space [7], [8], is important for understanding incidental perception and learning. The current study examined the recognition memory of younger and older adults for objects that they were incidentally exposed to while en route to a testing room. We evaluated recognition memory for three types of objects: context-typical objects, context-atypical objects, and novel unfamiliar objects with no known associated context.

The concept of *typicality* has been used to explain the influence of knowledge-structures known as schemas on processing visual stimuli. *Schemas* provide a set of expectations about the world based on prior experience [9]. Evidence suggests that objects are organized in memory within structures that depict typical scenes, and that these perceptual frames of knowledge [10] play an important role in memory functioning. In particular, participants often show especially good memory for items that are atypical or that violate schematic expectations [11]–[14], mainly because inconsistent items are visually fixated or processed longer than are consistent ones [15], [16]. However, participants also often falsely recall and/or recognize schema-consistent items that were not actually part of the original episode, and these memory errors are usually accompanied by high confidence or vivid recollection [4], [6], [11], [17], [18], for review, see [19]. As developed further below, these findings underscore the importance of adopting a testing format that minimizes differential contributions of response bias to memory judgments for different object types (e.g., for context-typical items).

**Figure 1:**
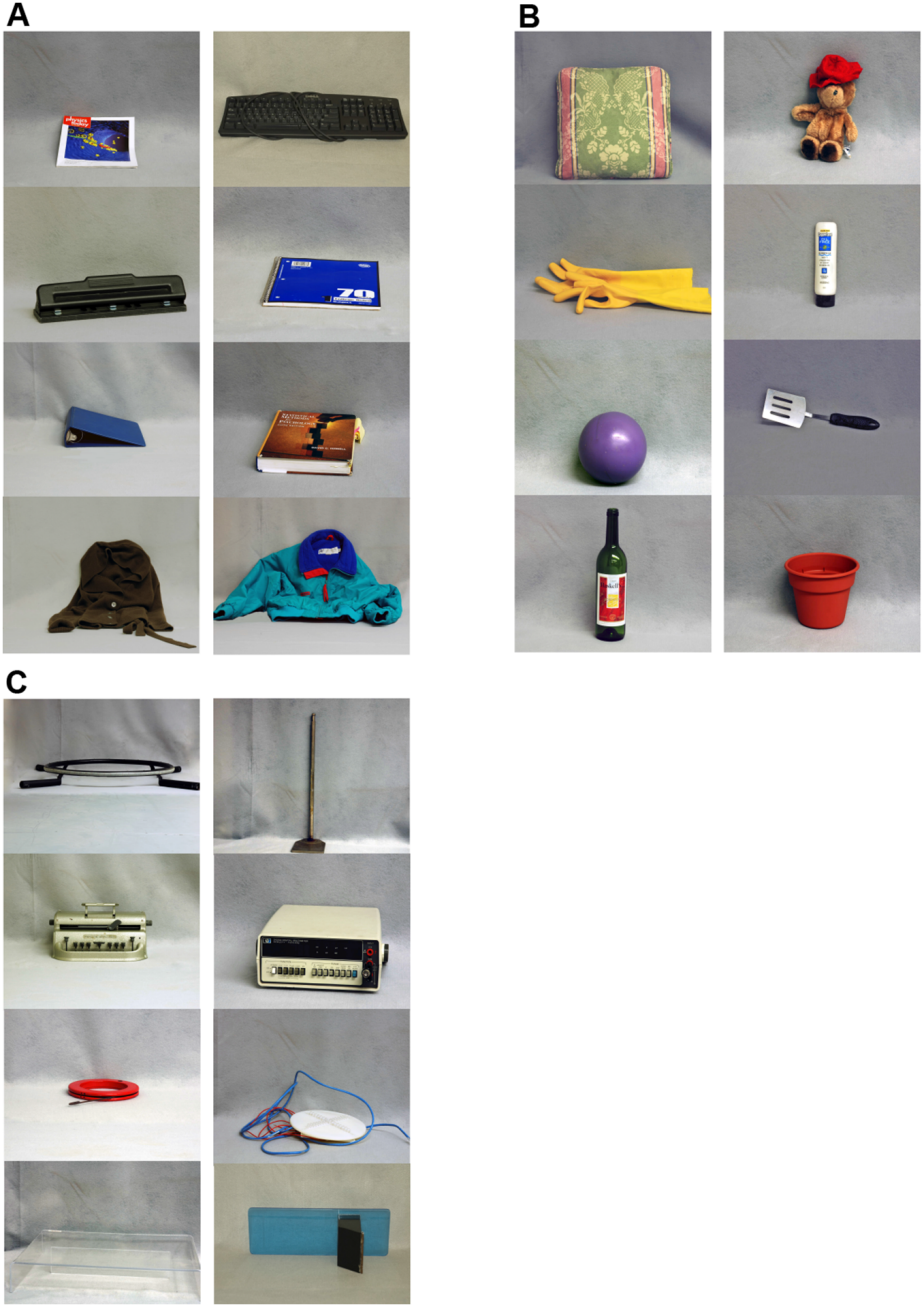
All objects used in the main experiment. (A) typical objects; (B) atypical objects; (C) unfamiliar objects. Left panel contains Set A objects; right panel contains Set B objects.

The present study compared older and younger adults’ incidental memory for the identity of objects arrayed in a larger conference room that they passed through on the way to a smaller testing room. Some of the objects were *context-typical* and others were *context-atypical* (i.e., objects that might commonly vs. rarely be encountered in that context, see Fig. 1A and 1B). Schema theory [9] and findings on schematic expectancy violations led us to predict that recognition accuracy would be better for the context-atypical objects than for the context-typical ones. Additionally, to further examine the effects of prior knowledge on incidental object memory, we presented a third group of *novel unfamiliar objects* that were not readily named and that had *no typically associated context* (see Fig. 1C). Because unfamiliar objects neither directly violate context expectancies, nor readily meet or fulfill them, they offer a major advantage. Studying them can aid in disentangling the effects of a *schematic expectancy violation* (as found in enhanced recognition of context-atypical objects compared with context-typical objects) and the effects of *integration*, or the incorporation of new episodic information into schema-based representations (as shown in elevated false memory for context-typical compared with context-atypical objects). If unfamiliar objects, like context-atypical objects, draw incidental attention, then they will be more accurately recognized than are context-typical objects. On the contrary, unfamiliar objects might be recognized less accurately than are context-atypical objects because they have no strong prior association with a particular situational context that might be violated. Thus, they might instead be integrated into the context. Given that they are unfamiliar, they may also be less readily named and processed [17], [20].

Older adults and younger adults have been found to show generally equivalent knowledge for schematic information, such as generic knowledge about routine activities [21], schematic spatial layouts [22], and other forms of knowledge [13], [23]. However, in tests of recognition using pictures (e.g., pictures of common objects versus abstract unfamiliar objects that have no known name or agreed-upon function) older adults show an elevated reliance on semantic category information [20]. The incidental memory encoding of older compared with younger adults for the novel unfamiliar real world objects was thus of particular interest, as these incidentally encountered objects (see Fig. 1C) provided little affordance for such reliance on semantics.

To maximize sensitivity to any incidental memory that participants retained from their brief traversal through the conference room, we used a two-alternative forced-choice (2AFC) recognition format, in which the presented memory test items were detailed color photographs of the objects. Additionally, to minimize differential contributions from response bias, target objects were always paired with a lure item from the same object-type (e.g., context-typical objects that had actually been presented in the conference room were paired with other context-typical objects that were not in the room). Across participants, items were counterbalanced across study and test status such that all items served equally often as targets and lures, and also occurred equally often at each of two distances (near or far) from the participant’s path through the conference room.

**Figure 2:**
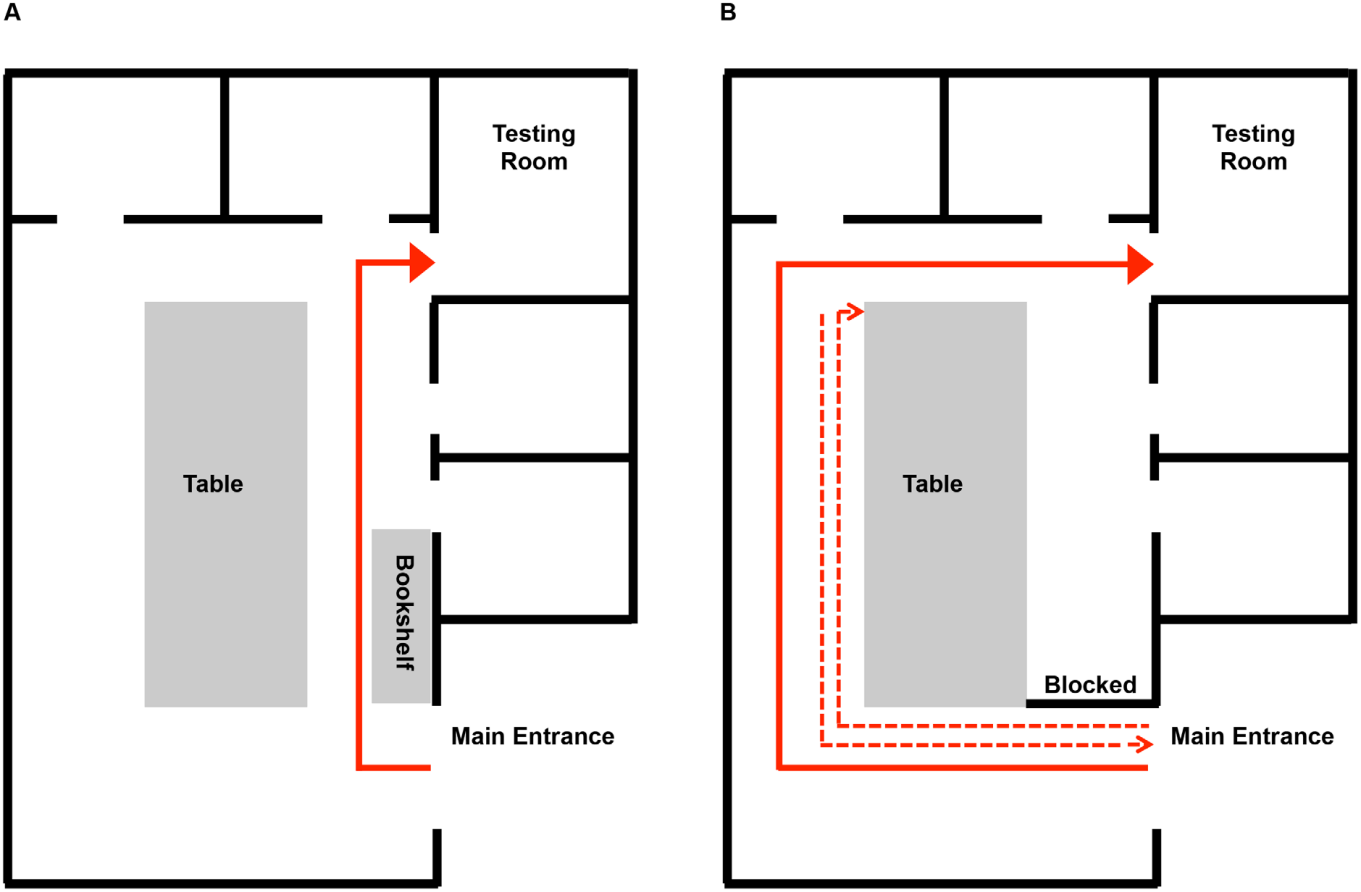
Map of the conference room. The solid arrows indicate the walking path from the door of the conference room to the testing room for the pilot study (A) and in the current experiment (B). In the pilot study, participants walked directly from the main entrance to the testing room. In the present study, participants first walked to the end of the table to retrieve the “experiment in progress” sign. Next, they returned to the main entrance and handed it to the experimenter, who was waiting there. Then they walked from the entrance to the testing room, traversing a longer route around the far end of the table because the shorter route was blocked with a bookshelf.

In a pilot experiment, 36 younger and 36 older adults were first met by an experimenter in a designated location, where they were provided background information and completed an informed consent process to take part in a larger multi-session study on “Attention, Memory, and Thinking.” The experimenter and participant then proceeded down a short flight of stairs toward the conference room. At the door to the conference room, the experimenter asked the participant to go on ahead to the testing room, which was one of several smaller sub-rooms leading off of the conference room, gesturing toward the testing room door, while the experimenter remained briefly behind to post a “testing-in-progress” sign on the outside of the conference room door. The participant then took a single unidirectional route from the entrance of the conference room to the testing room (see Fig. 2A). On the participant’s first visit to the lab, there were a few context-typical control objects (e.g., a coffee mug) on the table, and the participant was not asked any questions about what they had observed. On the participant’s second visit to the lab, a few days later, the same procedure was followed, but this time, the conference room table and a near-by bookshelf had an intermixed array of objects, including 6 context-typical, 6 context-atypical, and 6 unfamiliar objects (see Table 1 for a listing of the objects). Immediately after reaching the testing room the experimenter closed the testing room door, and administered a self-paced computerized 2AFC recognition test for the objects that had been present in the conference room. Participants were shown pairs of photographs of objects (e.g., the photographs of two context-typical objects, or of two unfamiliar objects), with each object displayed against a gray background (see Figure 1), and were asked to choose which one of the two objects in each pair had been in the conference room that they had just walked through. We found that older adults showed significantly above-chance levels of incidental recognition for both the context-atypical and the unfamiliar objects (recognition levels of approximately 60% for both) but they did not recognize context-typical objects at above the 50% expected on the basis of chance responding. The incidental recognition performance of younger adults was uniformly low and did not exceed chance levels for any of the object types.

**Table 1:** Two sets of stimulus objects.

No.	Set A	No.	Set B
101	*CD disc-holder*	501	Keyboard
102	Magazine (physics)	502	*Journal paper*
103	3-hole punch	503	Blue notebook
104	*Mug*	504	Textbook
105	Blue binder	505	*3M post-it-note*
106	Brown sweater	506	Blue jacket
107	Cushion	507	Teddy bear
108	*Shower cap*	508	Sun block
109	Rubber gloves	509	*Gardening sheers*
110	Purple ball	510	*Bead shaker*
111	*Chopping board*	511	Spatula
112	Wine bottle	512	Plant pot
113	Huge ring	513	Tall stand
114	Tactile typewriter	514	Electric equipment
115	Round red metal ring	515	*Silvery metal structure*
116	*Blue wax cut-out shape*	516	*Chin rest*
117	Transparent stand	517	Blue wires + white sphere
118	*Gold metal configuration*	518	Blue plexiglass

Objects in italics were used in the pilot study but not in the main experiment.

What might account for the numerically superior incidental recognition performance of the older adults in this pilot study? For several reasons, it was not entirely unexpected that the overall performance of older adults on the recognition task (for context atypical and typical objects) might prove to be at least somewhat similar to, or equivalent to, that of younger adults. In general, age-related memory deficits tend to be less pronounced under incidental encoding than under intentional encoding, because incidental encoding may be more automatic and less effortful [24]. The 2AFC test format minimized the influence of possible age differences in recognition criteria, such as a general tendency for older adults to respond “yes” [25], [26] or to respond on the basis of general semantic or gist information [27], [28], because the objects presented on each trial were from the same object type (e.g., both objects might be context-typical) and participants would have to find a reason for choosing one of those two objects. The 2AFC format with photographs of the objects also provided stronger retrieval support than do other testing formats, such as free or cued recall, that may prove more challenging for older than younger adults by placing greater demands on effortful retrieval and self-directed search [29]. From a broader perspective, older and younger adults show generally equivalent knowledge for schematic information, such as generic knowledge about routine activities [21], schematic spatial layouts [22], and other forms of knowledge [23]. But why might older adults have shown above-chance recognition, whereas younger adults did not?

One possibility is that older adults simply walked more slowly to the testing room, and so had more time to notice and encode the objects. However, our assessments of walking times in the pilot study (for 21 participants) indicated that older and younger adults seemed to take similar amounts of time in walking from the conference room door to the testing room, with the traversal to the testing room requiring an average of about 9.8 sec (*SD* = 0.58). Another possibility is that older adults’ attention was more often spontaneously drawn to the objects. In this study, the participants were asked to proceed ahead of the experimenter to the testing room and, from this perspective, walking toward the testing room might be seen as the participant’s “top-down” goal. If younger adults showed only chance-level incidental recognition for the objects, perhaps this was because of their stronger or more exclusive focus on that goal. Perhaps older adults were more generally alert to their relatively more novel surroundings or were more likely to be distracted by, and so pay attention to, goal-irrelevant information on their way to the destination (i.e., the testing room) than were younger adults.

If so, such distraction might be more likely to occur for context-atypical objects that violated normal situational expectancies, or for novel and unfamiliar objects than for context-typical objects. Compared with younger individuals, the task-related processing of older adults may be more susceptible to distracting information. This effect is sometimes characterized as an *age-related reduction in distraction regulation*, or as a deficit in the top-down suppression or inhibition of task-irrelevant information [30]. According to such an account, aging may be accompanied by changes in inhibitory attention mechanisms that help to dampen the activation of task-irrelevant thoughts or representations–including task-irrelevant thoughts that can be provoked by the presence of irrelevant stimuli in the task environment [31]. Increased sensitivity to distraction in older compared with younger adults has been shown across a variety of tasks. Among those tasks are reading [31], [32], problem solving [33], [34], and selective attention tasks involving the simultaneous presentation of task-relevant and task-irrelevant information, as when words are superimposed on irrelevant pictures, and attention should be directed to only the words [35], or superimposed face/place images with the task requiring attention to faces only [36].

Although our pilot results were generally in line with an age-related distraction account (if finding one’s way to the testing room in response to the experimenter’s request was considered a primary goal), and perhaps further suggested that context-atypical and unfamiliar objects might elicit similar levels of recognition performance, the low and either near-chance or at-chance levels of recognition in both age groups precluded strong conclusions. In the study reported here, we modified the experimental paradigm in several ways to elevate the recognition test performance of participants to above-chance levels and to enhance experimental control. We substantially reduced the number of target objects displayed in the room (from 18 to 12 items, see Table 1 for the included items, and Figure 1 for photographs of each of the objects), and placed all objects on, or directly next to, a large centrally located table. As detailed further below, we also modified the walking route that participants took en route to the testing room to a multiple paths route (see Fig. 2B) such that they had more time and greater opportunity to incidentally encode the objects, and we developed a procedure that equated the total amount of time in the conference room for all participants. Finally, we administered a brief post-experimental questionnaire to probe the extent to which participants surmised that their memory for the objects might be tested, allowing us to evaluate the possible effects of test awareness on recognition performance.

According to the age-related reduced distraction regulation account, older adults should show higher recognition for the incidentally encountered objects than younger adults. Also, according to schema theory, both age groups should show higher recognition for the context-atypical than for the context-typical objects. Recognition levels of older versus younger adults for the unfamiliar objects were of particular interest. Whereas context-atypical items violate schematic expectations and so may draw visual attention, unfamiliar objects do not have strong associated contexts and so may be less likely to attract attention and may be more likely to be integrated into the context. Unfamiliar objects are also comparatively novel, with little accompanying semantic information, and so may be less readily remembered. This may be especially true for older adults, who tend to rely more extensively on semantic or categorical information [20], [27], [37]. Nonetheless, our pilot results suggested that perhaps age-related distraction also might lead to strong encoding of the unfamiliar objects, such that recognition of these items by older adults would similarly be elevated above that for context-typical items.

## Methods

### Participants

Participants were 97 native-English-speaking young adults (*M* age = 19.81 years, *SD* = 1.94; 58 female) and 33 native-English-speaking older adults (*M* age = 69.24 years, *SD* = 5.14; 25 female), recruited at the University of Minnesota and from the Twin Cities community. All participants were screened for depression with the Brief Symptom Inventory [38] and for medical conditions that could affect their cognitive performance, and reported normal or corrected-to-normal vision. The Mini-Mental State Exam (MMSE), [39], was used to screen for the cognitive state of the older adults, and only individuals who scored 27/30 or higher were included (*M* = 28.94, *SD* = 1.30). The experiment was conducted following procedures approved by the University of Minnesota Institutional Review Board. Eight additional younger adults participated but were excluded from analyses: two had high depression scores (greater than 11), four participants’ data were missing due to computer problems, two participants were mistakenly tested in the incorrect counterbalancing conditions. Two additional older adults took part but their data were missing due to computer problems.

Younger adults on average had fewer years of formal education (*M* = 13.98, *SD* = 1.36) than did older adults (*M* = 17.39, *SD* = 2.21), *t*(128) = 10.48, *p*<0.001. Both older and younger adults rated their subjective state of health (7-point Likert scale, 1 = *very poor*, 7 = *excellent*) as close to excellent, without differences between the two groups (older: *M* = 6.03, *SD* = 0.05; younger: *M* = 5.75, *SD* = 0.95). Participants gave written consent to take part in the experiment and were compensated $10/hr.

### Materials

Participants passed through a conference room (10×5 m) en route to a testing room. The conference room (schematically diagrammed in Fig. 2) contained a large table, chairs and other fixtures. Two sets of 12 stimulus objects were used in this study (see Fig. 1 for photographs of all 24 stimuli). Depending on their familiarity and typicality in the context of a conference room, objects were classified into 3 groups: *context-typical* (e.g., a textbook), *context-atypical* (e.g., rubber gloves), and *unfamiliar* (i.e., unusual objects that were not associated with any typical context and that could not be readily named or categorized).

To confirm that the objects were appropriately classified, 6 older and 6 younger participants were asked to name each of the 24 objects and then indicate their confidence in the reported name on a 7-point Likert scale (1 = *very unsure*, 7 = *very sure*). They also rated the *familiarity* of each object (“How often do you encounter the object in your daily life?” 1 = *never*, 7 = *every day*), and the *context typicality* of each object (“How typical do you consider the object in the context of the conference room you have just walked through?” 1 = *not typical at all*, 7 = *very typical*).

Table 2 presents the descriptive statistics for these questions. Ratings from younger and older adults were highly correlated (*r* = .83, .85, and .89 for confidence in naming, familiarity, and context-typicality ratings respectively; all *p*<0.001). We combined younger and older adults’ data in the following analyses, treating object type as the unit of analysis. Confidence ratings in the reported names were higher for the context-typical and the context-atypical objects than for the unfamiliar objects, all *ts*(14)>8.98. Familiarity was higher for context-typical and context-atypical than for unfamiliar objects, all *t*s(14)>6.75. Context-typicality was higher for context-typical objects than for context-atypical or unfamiliar objects, *t*s(14)>8.35, and also for context-atypical objects than for unfamiliar objects, *t*(14) = 3.90.

**Table 2:** Mean naming confidence, familiarity, and context typicality ratings.

Naming Confidence	Older (*n* = 6)	Younger (*n* = 6)	Across age-group
Context-typical	6.71 (0.21)	6.79 (0.23)	6.75 (0.22)
Context-atypical	6.79 (0.15)	6.54 (0.63)	6.67 (0.46)
Unfamiliar	2.73 (0.89)	4.13 (1.46)	3.43 (1.37)
**Familiarity**			
Context-typical	4.44 (1.35)	5.60 (0.53)	5.02 (1.16)
Context-atypical	4.52 (1.23)	4.33 (1.04)	4.43 (1.10)
Unfamiliar	1.48 (0.33)	1.96 (0.50)	1.72 (0.48)
**Context Typicality**			
Context-typical	5.31 (0.86)	5.67 (0.61)	5.49 (0.74)
Context-atypical	3.40 (0.66)	2.40 (0.56)	2.90 (0.78)
Unfamiliar	1.98 (0.73)	1.81 (0.54)	1.90 (0.63)

Ratings for naming confidence, familiarity, and context typicality for context-typical, context-atypical, and unfamiliar objects for two groups of older and younger adults (standard deviations in parentheses). Ratings were made on a 7-point scale, with higher values indicating greater naming confidence, greater familiarity, and greater context-typicality.

**Figure 3:**
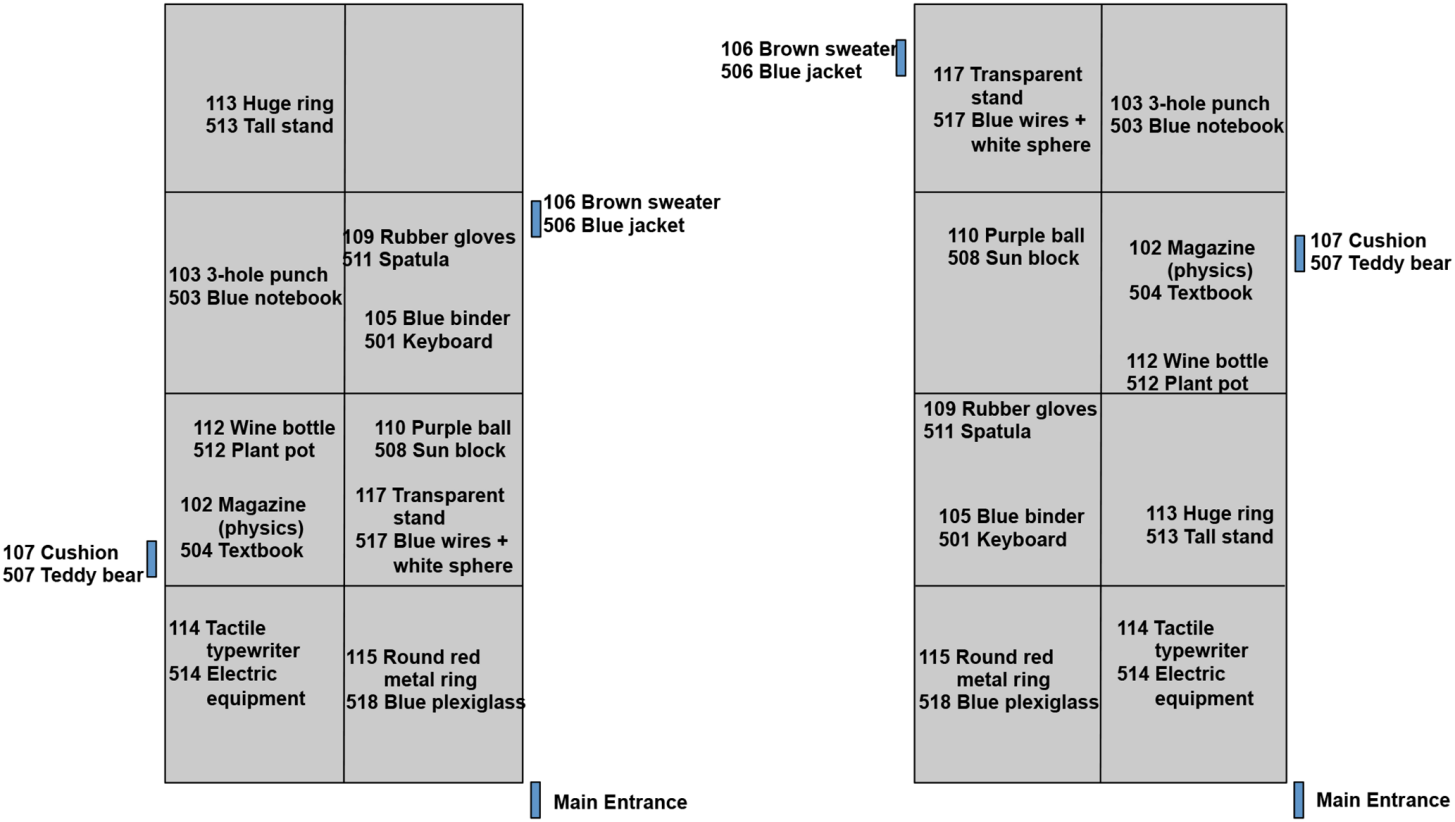
Object layout for the two sets in two counterbalanced maps. Left panel: map 1; right panel: map 2. Each participant only saw one set of objects on the table.

**Figure 4:**
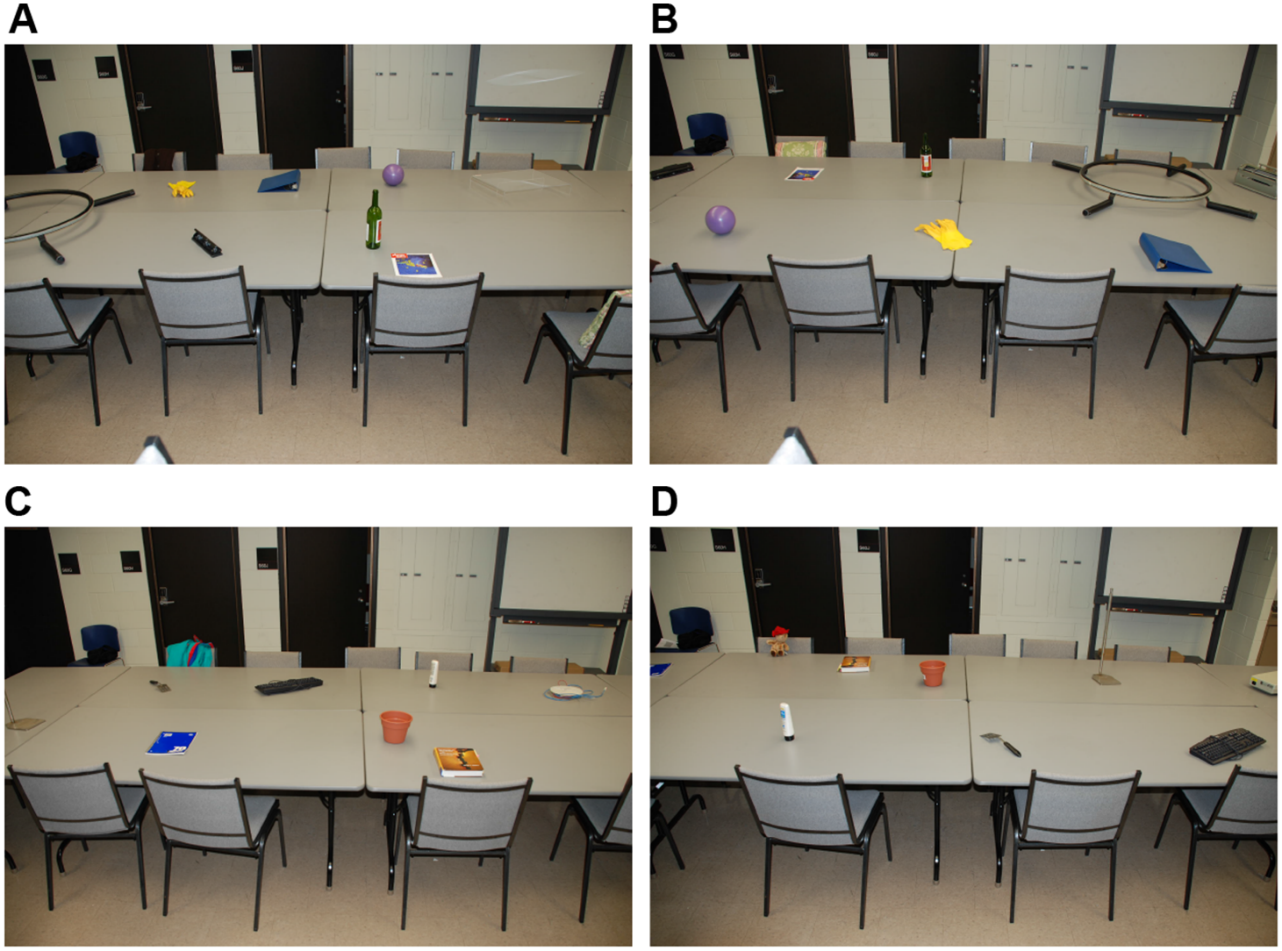
Example room photos for counterbalanced object set and map. (A) Set A–map 1; (B) Set A–map 2; (C) Set B–map 1; (D) Set B–map 2.

Four different object layouts (see Fig. 3 and Fig. 4) were created to counterbalance object presentations across studied status (presented or not) and distance (near or far). All objects were placed on or directly adjacent to a large table (5×2 m) to the right of the participant’s route to the testing room (see Fig. 2). Two items of each object-type were placed in the far area and two were placed in the near area. In addition to these critical experimental objects, other noncritical objects resided in the conference room (e.g., chairs, blackboards, and recycling bins).

### Procedure

Each participant was tested individually. Since age-related differences in cognition can vary according to the time of testing and participants’ self-reported time-of-day preference [34], participants selected their own preferred times to be tested. An experimenter met the participant in the main lobby area of the building, where the experimenter first obtained written informed consent for an experiment on “Attention, Memory, and Thinking”. The experimenter then escorted the participant to the conference room door. Upon opening the door the experimenter said, to the participant, “*Uh-oh, I forgot the Experiment-in-Progress sign, please help me to get it from the far end of the table in the room while I’m holding this door*”. The experimenter then gestured toward the sign on the table, drawing the participant’s attention to both the sign and the objects on the table. A second experimenter (inside the testing room, and hidden from the participant’s view) started a stopwatch for 100 seconds once s/he heard the experimenter say “*Uh*-*oh*”. The nearest route from the entrance door to the testing room was blocked to require participants to walk a longer route around three sides of the table (see Fig. 2B). After the participant retrieved the sign and gave it to the experimenter, the experimenter then said, “*Please go ahead to the testing room while I put up this sign indicating that an experiment is in progress*”. After putting up the sign, the experimenter entered the conference room.

An arrow sign at the far end of the conference room clearly instructed participants how to find the testing room. The testing room door was locked, with a note on the door stating that the room was in use by another experimenter who was running a little late but would be finished very soon. After a total of 100 seconds had passed, the second experimenter came out of the testing room and both the participant and the primary experimenter entered the room, closed the door, and started the memory test.

We used 100 seconds as the exposure duration because it was sufficient time for everyone (including older adults) to finish retrieving the sign and to see the note on the testing room door. Even the slowest participants had a little time left after they saw the note to wait for the experimenter inside the testing room to finish. While the participant waited, the experimenter walked along the table and pushed in 8 chairs (4 on each long side, placed at least 2 inches away from the table, but not impeding the walking path). By doing this, we hoped to standardize the interactions between the experimenter and the participant, while also subtly drawing the participant’s attention toward the table and objects so as to indirectly bolster incidental encoding of the objects to above-chance levels of recognition. If any time remained, the experimenter took out a large appointments schedule and pretended to be occupied with it. This discouraged participants from engaging in conversation with the experimenter and helped to equate the opportunities of participants to direct their attention to the room and the objects. Through this procedure, participants traversed the conference room three times (the first time for retrieving the experiment-in-progress sign from the far side of the table, the second time for coming back and giving the sign to the experimenter, and the third time for traversing the room to find and enter the testing room) before arriving in the final testing room. In addition, the exposure time to the objects was increased from approximately 9.8 seconds (in the pilot study) to 100 seconds.

Incidental memory for the 12 objects was assessed using a computerized, self-paced, two-alternative forced-choice recognition test. For each of the 12 trials, a photograph of the target (presented) object was paired with a photograph of one randomly selected, non-presented object from the same object-type (context-typical, context-atypical, or unfamiliar). The two alternatives were displayed side by side. Participants were asked to indicate via mouse-click which of the two photographs was the object that they had seen in the conference room. Participants were instructed to focus their attention on the object, rather than its appearance in the photograph (e.g., apparent size or distance), and to guess when unsure. Prior to testing, participants completed three practice trials on non-critical objects also previously encountered in the conference room (e.g., chair). The conference room was out of sight during testing, with the testing room door completely closed.

Directly after completing the recognition test, participants completed a brief post-experimental questionnaire regarding their naivety to the memory test before they had entered the testing room. Three key questions were embedded among filler questions: *“I suspected that I would be tested on the identities of the objects on the table”; “I made an effort to memorize the identities of the objects on the table as I walked through the room”; “If you made an effort to memorize the objects on the table, did you use a specific strategy to remember them?”* Participants answered the first two questions on a 5-point scale, labelled as *strongly agree, agree, neutral, disagree*, and *strongly disagree*. If participants answered “disagree” or “strongly disagree” on the first two questions and “no” on the third question they were classified as naïve to the recognition memory test. These participants were assigned a naivety score of 0 (naïve); all other participants were assigned a naivety score of 1 (non-naïve).

## Results

The average correct recognition rate for context-typical, unfamiliar, and context-atypical objects, respectively, was .81, .73, and .87 for older adults, and .69, .77, and .80 for younger adults. Independent *t-*tests were conducted to determine whether correct object recognition exceeded the proportion correct expected by chance (0.50). Recognition accuracy was significantly above chance, for all three object types for older adults, *ts*(32)>5.81, Cohen’s effect size *d*>2.00, *p*<0.001, and for younger adults, *ts*(96)>8.06, Cohen’s *d*>1.60, *p*<0.001.

Results from the post-experimental questionnaire revealed that, according to their answers to the three questions outlined earlier, 27 of the younger adults (28%) and 7 of the older adults (21%) were classified as non-testing-naïve. To examine whether participants’ self-reported expectations of recognition testing for the objects influenced performance, we correlated recognition for the three object types with participants’ naivety scores (scored 0 or 1 for naïve and non-naïve, respectively) from the post-experimental questionnaire. Naivety scores were modestly but significantly positively correlated with recognition of the context-atypical objects (point-biserial correlation, *r* = .21, *p*<0.05) and unfamiliar objects (*r* = .25, *p*<0.01) and showed a trend toward a positive correlation for context-typical objects (*r* = .16, *p* = 0.06). Additionally, across all participants, 2AFC recognition performance for the context-typical and context-atypical objects was significantly positively correlated (*r* = .37, *p*<0.001), but neither context-typical (*r* = .05) nor context-atypical (*r* = .15) object recognition performance was correlated with recognition of unfamiliar objects. A nearly identical pattern was observed for testing-naïve participants alone, when all non-naïve participants were excluded from the analysis (*r* = .36, *p*<.001 for context-typical with context-atypical, *r* = .003, and *r* = .15 for the correlations with unfamiliar objects). Although older adults, on average, had more years of formal education than did younger adults, years of education was not significantly correlated with object recognition performance either for the entire sample (*r* = .13, *ns*), or for testing-naïve participants alone (*r* = .11, *ns*).

**Figure 5:**
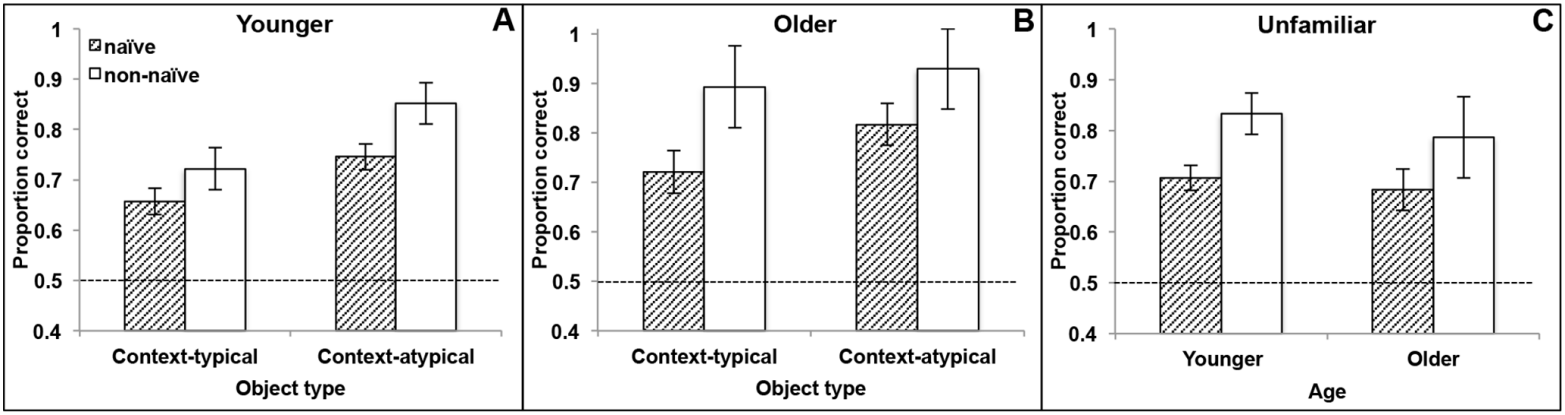
Mean recognition accuracy. (A) (B): Accuracy for context-typical and context-atypical objects for younger and older adults, shown separately for testing naïve and non-naïve participants; (C): accuracy for unfamiliar objects for younger and older adults, shown separately for testing naïve and non-naïve participants. Bars show standard errors of the mean; dashed line indicates chance performance.

Performance on the 2AFC object recognition test for the two age groups (younger and older adults) is shown in Fig. 5A and 5B respectively, separately by object type (context-typical, context-atypical) and test naivety (testing naïve, or non-testing naïve). As can be seen from the figure, recognition performance clearly varied by object type and also by test naivety, with the performance of older and younger adults apparently differing by object type.

To examine the object typicality effect, we first performed a 2(age group)×2(testing naivety–with naivety as a nominal grouping factor)×2(object type: context-typical or context-atypical) mixed-factor analysis of variance (ANOVA) on recognition rates for these object types. This analysis revealed a main effect of object type, with context-atypical objects (*M* = .84) being better recognized than typical (*M* = .75) ones – that is, a typicality effect, *F*(1,126) = 8.48, *p* = .004. There was also a significant effect of testing naivety, *F*(1,126) = 7.04, *p* = .009, with naïve participants (.74) scoring lower than non-naïve (.85), and also a significant main effect of age, *F*(1,126) = 5.00, *p* = .027, with older adults (.84) outperforming younger adults (.74), and demonstrating a 10% aging-related advantage. Neither age group nor test naivety interacted with object type, *F*s<1. For atypical objects, both older and younger test-naïve participants scored .11 lower than their corresponding non-naïve participants; for typical objects, older testing-naïve participants scored .17 lower, whereas younger testing-naive participants scored .07 lower. It is possible that, with greater statistical power, testing awareness would be associated with differentially larger recognition gains for older individuals specifically for typical objects.

Fig. 5C presents the object recognition results for the unfamiliar objects that had no strongly associated context for the two age groups, separately for the testing naïve and non-testing naïve participants.

We next separately examined the effect of age and test naivety for the unfamiliar objects that had no strongly associated context. A 2(age group)×2(test naivety) between-subjects ANOVA showed only a main effect of test naivety, with test naïve participants (*M* = .70) recognizing fewer of the unfamiliar objects than did participants who reported that they anticipated memory testing (*M* = .81), *F*(1,126) = 5.06, *p* = .026. There was no effect of age, *F*<1, with older (*M* = .73) and younger (*M* = .77) adults performing similarly for these unfamiliar objects, and no age×testing naivety interaction, *F*<1, with an essentially equivalent naivety difference of .11 for older individuals and .12 for younger adults.

A potential concern with these reported analyses is that the sample sizes for older and younger adults differed substantially, and there were also unequal numbers of participants who were classified as testing naïve versus non-testing naïve within each age group. To address these concerns, we also performed nonparametric analyses to assess the effects of object type, age, and testing naivety, in analyses that make fewer assumptions about the nature of the underlying distributions (e.g., using ranks and medians rather than means) and that are more robust to differences in sample size.

We began by examining the effects of object type (context-typical, context-atypical, and unfamiliar) across all participants, and then for test-naïve participants only. Related-samples Friedman’s two-way analysis of variance by ranks rejected the null hypothesis that the distributions of recognition scores across the three object types were the same, both for all participants, regardless of testing-naivety, *p*<.001 (*M* = .70, .79, and .73 respectively), and for testing-naïve participants alone (*M* = .67, .77, and .70 respectively), *p* = .003.

We next examined effects of testing-naivety and of age group. Independent samples Mann-Whitney U-tests showed that testing-naivety was again associated with significantly lower recognition scores overall (*M* naïve = .71, *M* non-naïve = .82, *p* = .001), with a similar pattern observed regardless of object type (context-typical objects: *M* naïve = .69, *M* non-naïve = .81, *p* = .09; context-atypical objects: *M* naïve = .78, *M* non-naïve = .89, *p* = .019; unfamiliar objects: *M* naïve = .70, *M* non-naïve = .81, *p* = .005).

Considering the average recognition performance for context-typical and context-atypical objects, an independent samples Mann-Whitney U-test again revealed a significant older adult advantage for all participants (regardless of testing naivety), *p* = .026; for these two object types, there was also a trend (*p* = .101) for the recognition performance of the testing-naïve older adults to exceed that of the testing-naïve younger adults.

Subsequent analyses focused on only the testing-naïve participants, with analyses performed separately for younger and older adults. Related samples Friedman’s two-way analysis of variance by ranks showed that there was a significant difference across the three object types (context-typical, context-atypical, unfamiliar) both for testing-naïve younger adults (*M* = .66, .75, .71, *p* = .041), and for testing-naïve older adults (*M* = .72, .82, and .68, *p* = .035). Excluding the unfamiliar objects, to specifically evaluate the typicality effect, showed a significant typicality effect for all naïve participants, *p* = .001, with the typicality effect also significant for testing-naïve younger adults alone, *p* = .011, and for testing-naïve older adults alone, *p* = .039. Furthermore, testing-naïve older adults showed significantly higher recognition for context-atypical objects than for unfamiliar objects, *p* = .04, whereas this pattern (of depressed recognition for unfamiliar relative to context-atypical objects) was not apparent in testing-naïve younger adults.

Finally, we examined the effects of distance, that is, objects that were near vs. far from the participant’s path through the testing room, on recognition for each of the three object types separately, using the non-parametric related samples Wilcoxon signed rank test. Testing-naïve younger adults showed no significant effects of distance for any of the object types (*M* near = .69, .74, and .73 for context-typical, context-atypical, and unfamiliar; *M* far = .64, .76, and .69, respectively). Testing-naïve older adults showed a tendency for higher recognition of near- than far-distance objects, particularly for context-atypical and unfamiliar objects, with this pattern marginally significant *(p* = .072) for the unfamiliar objects (*M* near = .73, .85, and .79 for context-typical, context-atypical, and unfamiliar; *M* far = .71, .77, and .60, respectively).

## Discussion

Although many investigations of object recognition have used computer-based presentations and two-dimensional stimuli such as photographs, more recent work has underscored the importance of examining visual attention and memory in natural environments and under natural viewing conditions [7], see also [5]. The current study examined incidental memory for objects encountered under comparatively more naturalistic viewing circumstances and during realistic goal-oriented movements through space in both younger and older individuals. Our modifications of the experimental procedures from those used in our initial pilot study were successful in boosting recognition performance to significantly above-chance levels in both age groups and for each of the three object types (context-typical, context-atypical and unfamiliar), thereby removing the floor effects in recognition and permitting examination of the typicality effect and the effects of object type on subsequent recognition memory for older versus younger adults.

The study has yielded four key findings. First, both older and younger adults demonstrated a typicality effect, showing significantly lower correct recognition for context-typical than for context-atypical objects. Second, when considering only the context-typical and context-atypical objects, and ignoring testing-naivety status, older adults showed significantly higher recognition performance for this non-goal-relevant information than did younger adults. This difference was no longer significant when confining consideration to only those participants who were testing-naïve, but older adults still numerically out-performed younger adults, with no evidence of an age-related impairment in incidental recognition. Third, the pattern of age-related performance differed for the *unfamiliar* objects with no strongly associated context. Whereas for younger adults, unfamiliar objects were correctly recognized more often than were context-typical objects (but slightly less often than context-atypical objects), for older adults recognition of the unfamiliar objects was lower than for the other two object types. For the testing naïve older adults, context-atypical objects were correctly recognized significantly more often than were unfamiliar objects. Additionally, although physical distance (near vs. far) from the participant’s main walking trajectory through the room had little influence on the performance of younger adults, regardless of object type, older adults tended to show somewhat higher recognition for objects that were nearer to them, with this pattern most apparent (though not significant) for the unfamiliar objects. Fourth, participants who retrospectively reported that they anticipated that their memory might be tested significantly outperformed those who did not report anticipating such testing. The test-awareness advantage was similar across the different object types and age groups, with no interactions of test-naivety with object type. We now focus our discussion on each of these four findings in turn.

### Typicality Effect in Older and Younger Adults

Our observation of a typicality effect in both older and younger adults is consistent with other findings suggesting that semantic memory functions are preserved–or even facilitated–with increasing age [20], [37], [40], [41]. Notably, we here observed a significant typicality effect even though: (a) we used a two-alternative forced-choice recognition test that included detailed color photographs of the targets and the lures, rather than a yes/no test or a verbal recognition test, and (b) the lures within each test trial were drawn from the same object type as the targets, thereby reducing the potential contribution of semantic inferences. For any pair of objects shown on the recognition test, participants could not simply say both were in the conference room, even if their schematic knowledge might have led them to believe both might well have been there; rather, they must choose one of the two objects. The provision of strong visual cues at test and the requirement for within-object-type discrimination (because targets were always accompanied by lures of the same object-type) may have made the recognition test more similar to a source recognition memory test. Source memory tests encourage reliance on, and closer analysis of and querying of any existing episodic memory, rather than reliance on semantic knowledge or inferences [42]. The two-alternative forced-choice testing format may have provided an especially strong means of tapping into any incidental information about the objects that participants retained, while discouraging excessive reliance (during testing) on semantic knowledge or inferences. The observation of a significant typicality effect, even under such strong retrieval support circumstances, is consistent with a substantial *encoding-related* contribution to the effect, as previously suggested by findings that inconsistent items are visually fixated or processed longer than are consistent ones [15], [16].

### Older Adult Recognition Advantage

Considering the context-typical and context-atypical objects, older adults demonstrated a recognition advantage over that shown by younger adults, with an average older adult memory advantage, across all participants (both testing-naïve and non-testing naïve), of approximately 10%. This outcome is consistent with the more tentative finding we observed in our pilot study where–under more difficult and briefer encoding conditions involving a single path through the conference room–the incidental object recognition of older adults for context-atypical and unfamiliar objects also numerically exceeded that of younger adults. However, the older adult recognition advantage was attenuated, and no longer significant, when analyses were confined to only the testing-naïve participants. This pattern suggests a possible important contribution of participant’s goals in determining incidental recognition.

One account of this finding is that, compared with younger individuals, the task-related processing of older adults may be more susceptible to distracting information. Reduced distraction regulation often leads to performance impairments–when the distracting information is not helpful, or detracts from an ongoing task. However, sometimes distracting information can prove beneficial, if what was previously irrelevant (or apparently irrelevant) becomes relevant [43], see [44] for a general review of the possible costs and benefits of an age-related increase in distractibility. A number of studies have yielded results in line with this suggestion. For instance, in one study [33], participants were presented with a reading task accompanied by distractor words. Unbeknownst to the participants, the distractor words were actually solution words to complex remote associate problems that they were–unexpectedly–later asked to solve. Whereas older and younger adults solved similar numbers of remote associate problems in the control condition, for which the solutions had not previously been presented, older adults outperformed younger adults for target problems for which the distracting words had presented a solution to an as-yet-never encountered remote associate problem.

Age-related differences in the initial processing of irrelevant or distractor information have also been observed in neuroimaging studies [36]. In one study [45], participants were shown alternating pictures of scenes and faces, and were asked to remember only the scenes and to ignore faces. Under these conditions, older and younger adults showed comparable levels of brain activity in the scene-selective parahippocampal place area for the (to-be-remembered) scenes. In contrast, when the instructions were to remember the faces and not the scenes, activation in the parahippocampal place area was reduced for the younger adults, but not for the older adults. Older adults also later rated the irrelevant “distractor” scenes as more familiar, suggesting differences in the extent to which they had processed the distracting information.

Another possible account of the age differences in incidental recognition focuses on the performance of the younger adults: rather than older adults being especially distractible, perhaps younger adults were especially unobservant. Indeed, distraction was neither explicitly manipulated nor measured in this experiment and, if we consider walking to the testing room as the participant’s primary top-down goal, then looking around the room would not necessarily be in conflict with that (comparatively non-demanding) goal, and might be construed as consistent with it.

Future research should further test the circumstances under which older adults show enhanced incidental recognition for objects in relatively novel environments. If consistently found, such enhanced recognition might speculatively serve the function of increasing the likelihood that older individuals would notice unexpected obstacles or challenges to their safe trajectory through an unfamiliar space.

### Recognition of Unfamiliar Objects

In contrast to many previous studies that have compared only context-typical versus context-atypical objects, in the current experiment we also included objects that were unfamiliar and that did not possess strongly associated contexts. Whereas younger adults appeared quite adept in recognizing these unfamiliar objects and demonstrated, on average, a level of correct recognition (.77) that was quite close to that for the context-atypical objects (.80), older adults showed a different pattern. For older adults, average correct recognition of the unfamiliar objects (.73) fell 14% below that shown for the context-atypical objects (.87), and testing naïve older adults showed significantly higher recognition for the context-atypical objects than for the unfamiliar objects that had no prior associated context. Tentatively, these results might suggest that whereas unfamiliar objects may have tended to elicit expectancy violations in younger adults, they were more likely to lead to integration with the context for older adults. Although the average context-typicality ratings for older and younger adults did not differ when they were asked to evaluate the context-typicality of the objects directly, as an explicit and focal task (see Table 2), age differences in integration (versus expectancy violation) might emerge when unfamiliar novel objects are incidentally encountered “in situ” during another task, with no direct task requirement for evaluation of their contextual appropriateness. Within-subject correlations for recognition across the three object types also suggested that the bases for recognition of the unfamiliar objects differed from both the context-typical and context-atypical objects. Whereas recognition of context-typical and of context-atypical objects was significantly positively correlated, object recognition performance for these two object types was only very weakly and non-significantly correlated with recognition of unfamiliar objects.

Future research might seek to better establish the bases for incidental recognition of novel objects that neither violate, nor are likely to be schematically integrated with, their surrounding context and also the reasons for an age-related difference in the incidental processing of such objects. Such research (particularly if combined with eye-tracking data) might focus especially on the role of context-violation versus item-novelty and semantic knowledge in age-related memory performance. This work could provide additional analytical leverage to examine theoretical accounts of the contextual typicality effect that emphasize the important role of the inconsistency of target items with the predominant schema or gist of a scene [14], [16]. For example, a recent study of change detection in younger adults found that whereas context incongruency facilitated the process of *detecting* and *localizing* the object that was changing, congruent contexts appeared to facilitate *identification* of the object that was changing [46]. Inclusion of novel unfamiliar items might add additional leverage here.

### Incidental Encoding of Objects and Test-expectancy Effects

In our pilot study, we observed chance levels of recognition performance for younger adults across all three object-types and chance level performance for older adults for the context-typical objects. This was so even though the recognition test provided strong retrieval support in the form of colored photographs and the two alternative forced-choice testing format additionally provided participants the opportunity to draw upon any existing familiarity difference between the two alternatives [47]. At a broad conceptual level these findings appear congruent with arguments that incidental learning and change detection are heavily goal-dependent [8], [48]. In our pilot experiment, young adult participants may have focused primarily on reaching the testing room with little incidental attention devoted to their surroundings en route to that destination, and little or no memory for the objects in the conference room.

In the experiment reported here, we sought to provide more “incidental” opportunities for the encoding of the objects, and were successful in bolstering recognition levels for all object types and for both age groups to significantly above chance levels. However, the elaborate procedure we adopted for extending the participant’s object encoding opportunities (including the request to retrieve the “experiment in progress” sign, such that participants traversed the room three times rather than only once) also led some participants to suspect that their memory for the objects in the conference room might later be tested. We observed slight to modest positive point-biserial correlations between object recognition performance and scores on the post-experimental questionnaire for test naivety (correlations between .16 and .25). However, the observed patterns of recognition performance across the object types did not interact with test-naivety. This suggests that even in participants who suspected a memory test, encoding of the objects was strongly influenced by the object’s relative fit or non-fit with the general situational context.

A previous study with younger adults using a task somewhat similar to ours found that incidental recognition memory for landmarks was quite good, and did not differ under intentional versus incidental encoding instructions [49]. It is worth noting though, in our paradigm, that the objects were not likely to act as “landmarks” for navigating to the testing room because all of the objects were placed directly on or adjacent to the large centrally located table, and the route to the testing room was both straightforward and clearly indicated with a posted arrow sign.

Across-age group effects in recognition memory were also examined in another recent study [50]. These researchers implicated the importance of the participant’s overall task goals in the pattern of age-related memory effects observed. In their study, the central task involved looking at a photograph of common objects placed in different positions in a testing room cubicle. They manipulated whether participants expected to be tested (intentional encoding) or not (incidental encoding) after viewing the photograph. However, in both cases, the participant’s focal goal and task orientation was looking at the photograph. Overall, younger adults showed higher recognition memory for the objects, and higher confidence in their chosen targets, than did older adults. Specifically, after viewing the photograph, participants were shown 24 actual objects, and were asked to select the 12 target objects that had earlier been presented in the photograph. Younger adults showed higher recognition than did older adults both for intentional encoding (.76 versus .71, respectively) and for incidental encoding (.73 versus .63, respectively). This study, and their results with respect to age-differences, substantially diverges from our paradigm, when the participant’s focal goal/task orientation was to navigate to the testing room, and there was no explicit task relevance regarding the objects. Thus, although older adults do often demonstrate decreased episodic memory, the extent to which age-related deficits are observed, and whether deficits emerge at all, depend on the actual task and paradigm used in different studies. While a substantial portion of the research literature on aging has pointed to declines in cognition and performance with age [51], the findings reported here for the context-typical and context-atypical objects clearly show that, at least under some circumstances, older adults may have equivalent (if not better) incidental memory than do younger adults.

### Limitations and Future Directions

While our study contributes to understanding the typicality effect during the natural viewing of scenes and to findings concerning age-related differences in incidental memory, (see [52], for evidence of the typicality effect in older adults under intentional encoding), we are aware of some limitations. Our aim of examining incidental recognition for familiar and unfamiliar objects in a real world context, together with the below-chance levels of recognition we observed in our pilot experiment, restricted the number of objects of each type that we could include. Given logistical difficulties in recruiting eligible older adult participants, we included a larger number of younger participants (*N* = 97) than older participants (*N* = 33). Our sample size for older participants may have limited our ability to detect more subtle age-related patterns such as a possible age×distance interaction for unfamiliar objects. Nonetheless, the outcomes from the nonparametric analyses point to the reliability of the central findings, especially those of object type, and testing naivety. Another potential limitation is that a global context effect may have contributed to the performance advantage for older adults. Young university students may be more familiar and at ease in navigating through a conference room in a campus building [15], whereas older adults, who have typically had less campus contact for many years, may be more focused on their surroundings and on navigating to the proper location. In short, because the global context (schema) may have been more familiar to younger adults, they may have been less attentive to the detailed surroundings, potentially accounting for their lower object recognition accuracy. This account is, in some respects, a variant of the differential goal-related processing account that we have posed here. Two points, however, partially counter this possibility. First, older adults also showed a recognition advantage in our pilot experiment–and in that experiment we tested incidental recognition on everyone’s *second visit* to the lab, after an earlier visit to the lab and to the same testing room; thus all participants had recently been exposed to the general context and the conference room itself. Second, a global context effect cannot readily explain the differential patterns across age groups for the three object types that we observed in the current study, particularly the observation that, for testing-naïve older adults, context-atypical objects were associated with significantly higher recognition than were unfamiliar objects that had no strongly associated prior context.

A final important limitation is a concomitant of our efforts to study incidental memory for objects encountered in the real world, as individuals are engaging on another task. This is simply that we had comparatively little control over where participants looked, or for how long, or to what other endeavors (e.g., checking their cell phones for messages) they may have briefly turned their attention during the 40 to 60 seconds they had remaining after retrieving the testing-in-progress sign for the experimenter, and subsequently finding the note on the testing room door asking them to wait. As noted, we developed the incidental exposure procedures that we did because, without them, the performance of younger adults remained at chance levels, precluding any examination of the influence of object type. We could and did develop ways to standardize the intervening time as much as possible from the experimenter’s point of view (e.g., the experimenter systematically pushed in the chairs around the table, and consulted a daily planner so as to discourage participants from differentially engaging in conversation with the experimenter). Our results thus must be interpreted as applying to situations that provide comparatively unstructured brief incidental encoding opportunities, in which individuals choose the manner in which they direct their attention to either their own affairs, or to more actively perceptually exploring and noticing their surrounding environment.

Future work is needed to address these limitations, to further tease apart the factors that contribute to incidental memory for objects in the real world, and to shed light on how these factors interact. One possible approach is to use a virtual reality environment. Although virtual reality paradigms may have less ecological validity than real world experiments, they may represent a good *compromise* between the competing requirements for increased experimental control, sufficient flexibility, and ecological validity. Using a virtual reality environment, one study [53] found no age impairments in incidental memory for items encountered and, indeed, under incidental encoding, item recall in older adults was numerically higher than in younger adults. Virtual reality environments might enable memory testing for a larger number of objects, enhancing generalizability, as participants could be exposed to several different spaces (e.g., office, park, etc.) before memory testing, if, for example, they were virtually moving through multiple different rooms in a building or in successive buildings. Virtual reality environments might also more readily allow experimental manipulation of the degree of pre-existing familiarity with the scenes, and with the objects themselves, including the contexts in which those objects have formerly been encountered [54].
